# Association of Physical Activity with the Risk of Hepatocellular Carcinoma in Patients with Chronic Hepatitis B

**DOI:** 10.3390/cancers13143424

**Published:** 2021-07-08

**Authors:** Ho Soo Chun, Sojeong Park, Minjong Lee, Yuri Cho, Ha Sung Kim, A Reum Choe, Hwi Young Kim, Kwon Yoo, Tae Hun Kim

**Affiliations:** 1 Ewha Womans University Medical Center, Department of Internal Medicine, Ewha Womans University College of Medicine, Seoul 07804, Korea; 01205s@eumc.ac.kr (H.S.C.); archoi20@ewha.ac.kr (A.R.C.); hwiyoung@ewha.ac.kr (H.Y.K.); yook57@ewha.ac.kr (K.Y.); 2 Data Science Team, Hanmi Pharmaceutical Co., Ltd., Seoul 05545, Korea; sojeong.park@hanmi.co.kr (S.P.); hasung@hanmi.co.kr (H.S.K.); 3 National Cancer Center, Center for Liver and Pancreatobiliary Cancer, Goyang 10408, Korea; yuricho@ncc.re.kr

**Keywords:** physical activity, chronic hepatitis B, hepatocellular carcinoma

## Abstract

**Simple Summary:**

Although viral replication in patients with a chronic hepatitis B (CHB) infection is effectively suppressed by potent antiviral therapy such as entecavir or tenofovir, the risk of hepatocellular carcinoma (HCC) development in CHB patients cannot be totally eliminated. Thus, control of modifiable risk factors for HCC development such as lifestyle modification is important to minimize the HCC risk. In this study, we analyzed a nationwide population-based cohort to evaluate whether there is a significant association between physical activity and development of HCC in CHB patients treated with entecavir or tenofovir. Results in this study suggest that physical activity was significantly associated with a lower risk of HCC development in CHB patients treated with potent antiviral therapy. Increasing physical activity can have beneficial outcomes on HCC development in CHB patients treated with entecavir or tenofovir.

**Abstract:**

Background and Aims: In the general population, previous studies reported that physical activity was associated with risk of hepatocellular carcinoma (HCC) development. However, it is unclear whether physical activity is associated with risk of HCC development in patients with chronic hepatitis B (CHB). We aimed to elucidate the association between physical activity and risk of HCC development in CHB patients. Methods: This nationwide cohort study involved treatment-naive patients with CHB (*n* = 9727) who started treatment with entecavir or tenofovir and answered self-reported questionnaires between January 2012 and December 2017, using data from the Korean National Health Insurance Service database. The primary endpoint was development of HCC. Multivariable Cox regression and competing risk analyses were used. Results: During a median follow-up of 3.1 years, cumulative HCC incidence rates were 8.3%. There was an inverse association between physical activity and the risk of HCC (*p* < 0.001). Patients with 1000–1500 metabolic equivalent task (MET)-min/week, compared to those without physical activity, showed a significantly lower risk of HCC in both patients without cirrhosis (adjusted hazard ratio [aHR] 0.66, *p* = 0.02) and patients with cirrhosis (aHR 0.61, *p* = 0.02). In patients who were younger (<60), male, without diabetes, and with high BMI, amounts of physical activity of 1000–1500 MET-min/week showed an inverse association with the risk of HCC (aHR 0.65, 0.63, 0.65, and 0.64, respectively, all *p* < 0.05). Conclusion: Physical activity was significantly associated with a low risk of HCC in CHB patients treated with entecavir or tenofovir.

## 1. Introduction

For patients with a chronic hepatitis B (CHB) infection, the role of nonviral risk factors in hepatocarcinogenesis is of particular important because the risk of hepatocellular carcinoma (HCC) development cannot be totally eliminated even after viral replication is suppressed by potent antiviral therapy such as entecavir or tenofovir [1,2,3,4,5,6,7]. Physical activity or exercise has also been considered to prevent sarcopenia, frailty progression, hepatic steatosis, or cardiovascular complications from other comorbidities. As patients with CHB age, they increasingly experience common comorbidities such as diabetes mellitus, hypertension, and other cardiovascular diseases [8,9,10,11,12,13,14].

The benefit of physical activity for the prevention of HCC was suggested by evidence from preclinical data. The recently-published literature which utilizes a mouse model with HCC induced by diethylnitrosamine suggested that exercise stimulates hepatic p53 activation, upregulating cell cycle inhibitor p27, suppressing cyclin E, and dampening activation of c-Jun N-terminal kinase signaling, thereby regulating hepatocarcinogenesis in mice independently of weight control [15]. In another study using HCC rats, regular physical activity increased phosphatase and tensin homolog deleted from chromosome 10 (PTEN) expressions and adenosine monophosphate-activated protein kinase (AMPK) phosphorylation while decreasing the phosphorylation of protein kinase B, S6 ribosomal protein, and signal transducer and activator of transcription-3 (STAT-3) [16]. Transcriptomic analysis further suggested that the major effects of exercise were on the nontumoral liver rather than tumor tissue, wherein exercise demonstrated similar beneficial effects when combined with sorafenib [16]. Moreover, in nonalcoholic steatohepatitis mouse models [17], regular exercise stimulated the phosphorylation of AMPK and its substrate raptor, which decreased mechanistic target of rapamycin (mTOR) kinase activity, an important signaling pathway involved in HCC carcinogenesis. Until recently, there were no data regarding association of physical activity with the risk of HCC development in high-risk patients with chronic hepatitis B or hepatitis B virus (HBV)-related liver cirrhosis, particularly patients who showed good compliance with potent antiviral therapy such as entecavir or tenofovir.

Thus, in CHB patients treated with entecavir or tenofovir, it is unclear exactly how much physical activity can be effective in further lowering the risk of HCC development in real practical fields. In this study, we analyzed a nationwide population-based cohort to elucidate whether there is a significant association between physical activity and development of HCC in CHB patients treated with entecavir or tenofovir.

## 2. Methods

### 2.1. Study Population

A national health claims database, the National Health Insurance Services (NHIS)–Health Screening Cohort was used in this study. Details about the cohort have been described previously [18,19]. Individuals in the insurance system aged 40 years or older are entitled to undergo a general health screening program every 2 years. The program includes self-reported questionnaires, blood pressure measurements, and laboratory tests. Standardized questionnaires are used to acquire information on previous medical history and lifestyle factors such as smoking, alcohol intake, and physical activity. Korea has a single-payer, universal health coverage system, and the NHIS provides health insurance to more than 99% of the population. The study protocol complied with the ethical guidelines of the World Medical Association Declaration of Helsinki and was approved by the institutional review board of the hospital. The requirement for written informed consent was waived because the NHIS database was constructed anonymously following strict confidentiality guidelines.

**Figure 1:**
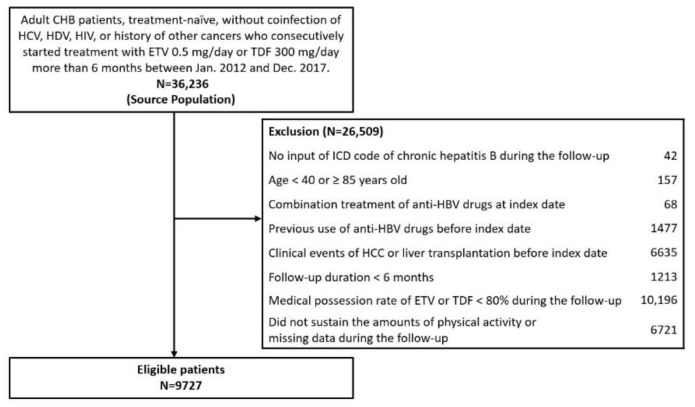
Flow diagram of the study population. Abbreviations: CHB, chronic hepatitis B; ETV, entecavir; HCV, hepatitis C virus; HIV, human immunodeficiency virus; TDF, tenofovir disoproxil fumarate.

Between 1 January 2012 and 31 December 2017, we extracted 9727 treatment-naive patients with CHB who started treatment with entecavir, 0.5 mg per day, or tenofovir disoproxil fumarate, 300 mg per day, underwent the screening program, and completed surveys on physical activity from the NHIS database historical cohort. All patients had International Statistical Classification of Diseases and Related Health Problems, Tenth Revision (ICD-10) code B18.1 for CHB. Cirrhosis was defined as ICD-10 code K74. Diagnostic codes used in the NHIS database are provided in Supplementary Table S1. Patients meeting one or more of the following criteria were excluded: aged younger than 40 years or 85 years or older at baseline; received a diagnosis of hepatitis C, hepatitis D, or HIV infection; received other treatments for chronic hepatitis B except entecavir or tenofovir; received a diagnosis of HCC or liver transplantation before the index date; had a follow-up duration shorter than 6 months after treatment; did not sustain the amount of physical activity during the follow-up based on a biennial NHIS health check-up data (no changes of the amount of physical activity among groups); or had less than 80% of medication possession rates (MPRs) of entecavir or tenofovir (Figure 1). To evaluate patient compliance with entecavir or tenofovir, we calculated the MPRs of the patients treated with entecavir or tenofovir stratified by specific therapy. The MPR values were calculated by dividing the total days of medication supply by the time interval. We used an MPR cutoff value for good compliance of more than 80% [20,21]. The reimbursement criteria for entecavir or tenofovir were identical and did not change during the study period: serum HBV DNA levels of 20,000 IU/mL or greater for patients positive for hepatitis B e antigen (HBeAg); 2000 IU/mL or greater for patients negative for HBeAg; and alanine aminotransferase (ALT) levels of 80 U/mL or greater in the absence of cirrhosis. In the presence of cirrhosis, the criterion was HBV DNA levels of 2000 IU/mL or greater [22,23].

### 2.2. Physical Activity

All subjects answered questionnaires about their daily leisure-time physical activity according to a previously described method [24,25]. Briefly, physical activity was measured using a modified Korean version of the physical activity questionnaire from the National Health and Nutrition Examination Survey, which employs a previously well-established metabolic equivalent (MET) quantification of physical activity [24,25,26,27,28,29]. Physical activity-related energy expenditure (MET-min/week) was calculated by summing the product of frequency, intensity, and duration. The level of leisure-time physical activity was categorized into 0 (totally sedentary), <500, 500–1000, 1000–1500, and ≥1500 MET-min/week.

The survey included three questions that addressed the usual frequency (days per week) of (1) light-intensity activity for at least 30 min (e.g., walking at a slow or leisurely pace), (2) moderate-intensity activity for at least 30 min (e.g., brisk walking, slow cycling, or tennis doubles), and (3) vigorous intensity activity for at least 20 min (e.g., jogging or running, bicycling >15 km per hour, climbing briskly up a hill, or participating in an aerobics class). Ratings of 2.9, 4.0, and 7.0 METs were assigned for light-intensity, moderate-intensity, and vigorous-intensity activities, respectively [30].

### 2.3. Clinical Evaluation and Follow-Up

The primary outcome was development of HCC. The development of HCC was defined by ICD-10 code C22 (Supplementary Table S1). The diagnosis of HCC using claims data in the NHIS database was previously validated to be highly accurate [31]. Death records from the Statistics Korea database were merged into the data set. The index date was the date when a patient first received a prescription for entecavir or tenofovir. The amount of physical activity close to the index date was used for analysis. The follow-up period for each patient was calculated from the index date to the date of HCC diagnosis, death, or the last follow-up (31 December 2018). Significant alcohol consumption was defined as consumption of ≥210 g of alcohol per week in men and ≥140 g of alcohol per week in women.

### 2.4. Statistical Analysis

The data were presented as mean ± standard deviations (SD) or median with interquartile (IQR) value for continuous variables and as number and percentage for categorical variables. Continuous variables were compared using a Student’s *t*-test or Wilcoxon rank-sum test, as appropriate. Categorical variables were compared using χ^2^-test with Yates’ correction for categorical variables. The incidence rate of HCC was calculated by dividing the number of deaths by the sum of the follow-up duration and presented as the rate per 100 person-years. Kaplan–Meier survival curves were constructed and compared using the log-rank test. Multivariable Cox proportional-hazard models were used to calculate adjusted hazard ratios (aHRs) and 95% confidence intervals (CIs). Drug exposure to medications, such as aspirin or statins, was considered as a time-dependent covariate to account for immortal time bias of waiting time for the individuals who were treated during the study period. In the time-dependent analysis, person-days of follow-up for the patients who received treatment after the index date were classified as untreated until the treatment started [32]. Multivariable Cox regression models were constructed with adjustment for age, sex, cirrhosis, diabetes, hypertension, body mass index (BMI), smoking, alcohol drinking, serum aspartate aminotransferase (AST), serum alanine aminotransferase (ALT), serum gamma-glutamyl transpeptidase (γ-GT), serum total cholesterol, serum creatinine, fasting glucose levels, entecavir or tenofovir, and the use of aspirin or statins (time-dependent variables). Subgroup analyses were done according to status of cirrhosis, age, sex diabetes, and BMI at baseline. Competing risk analysis was conducted for the interpretation of the cumulative incidence of HCC using Gray’s method. Death and liver transplant were defined as competing events for HCC [33,34]. *p*-Values of less than 0.05 were considered statistically significant. All statistical analyses were performed with SAS software version 9.4 (SAS Institute, Cary, NC) and R programming version 3.3.3 (http://www.R-project.org, 12 January 2021); the R Foundation for Statistical Computing, Vienna, Austria).

## 3. Results

### 3.1. Baseline Characteristics

A total of 9727 patients (mean 49 age; 6008 men [61.8%)] took part in this study. The baseline characteristics of these patients are shown in Table 1. A total of 2125 (21.8%) had cirrhosis, 581 (6.0%) had hypertension, and 404 (4.2%) had diabetes. The median value of body mass index was 24.0 kg/m^2^, serum ALT levels were 61 IU/L, serum low-density lipoprotein cholesterol levels were 108.0 mg/dL, serum creatinine levels were 0.9 mg/dL, and serum fasting blood glucose levels were 93.0 mg/dL. The median value of physical activity was 454 MET-min/week. In the study population, 4972 (51.1%) had physical activity values less than 500 MET-min/week, that is, they failed to achieve the recommended physical activity level (≥500 MET-min/week) in the general guidelines.

During the median follow-up duration of 3.1 years (IQR, 2.0–4.6 years), HCC developed in 809 (8.3%) patients. The median treatment duration of entecavir or tenofovir was 3.0 years (IQR, 1.9–4.4 years). Compared to those without HCC, patients with HCC were significantly older, male-predominant, smokers, consumed significantly more alcohol, had higher BMI, a higher proportion of diagnosis with liver cirrhosis and diabetes at baseline, and lower amounts of physical activity. Compared to those without cirrhosis, patients with cirrhosis were significantly older, had higher BMI, and a higher proportion of diagnosis with diabetes at baseline (Table 1).

There was no significant difference in the amount of physical activity between patients with cirrhosis and patients without cirrhosis (*p* = 0.10; Table 1 and Supplementary Figure S1). The proportion of patients who were sedentary was 1435 (18.9%) in the group without cirrhosis and 455 (21.4%) in the group with cirrhosis. When the proportions of physical activity were classified into five categories stratified by sex, the results suggested that there was a significant difference in the proportions of physical activity between the two groups (*p* < 0.001): male patients had more physical activity than female patients (Supplementary Figure S2).

### 3.2. Association between Physical Activity and the Development of HCC

A total of 809 patients (8.3% of 9727 patients) developed HCC during the follow-up period. In multivariable Cox regression and competing risk analyses, the results suggested that patients with a physical activity level of <500 MET-min/week (adjusted hazard ratio (aHR) 0.82; 95% confidence interval (CI), 0.68–0.99; *p* = 0.04), those with a physical activity level of 500–1000 MET-min/week (aHR 0.74; 95% CI, 0.61–0.89; *p* = 0.002), those with a physical activity level of 1000–1500 MET-min/week (aHR 0.64; 95% CI, 0.50–0.84; *p* = 0.001), and those with a physical activity level of ≥1500 MET-min/week (aHR 0.70; 95% CI, 0.51–0.97; *p* = 0.03) were at a significantly lower risk of HCC development compared to those who had a totally sedentary lifestyle (Table 2 and Supplementary Table S2).

**Figure 2:**
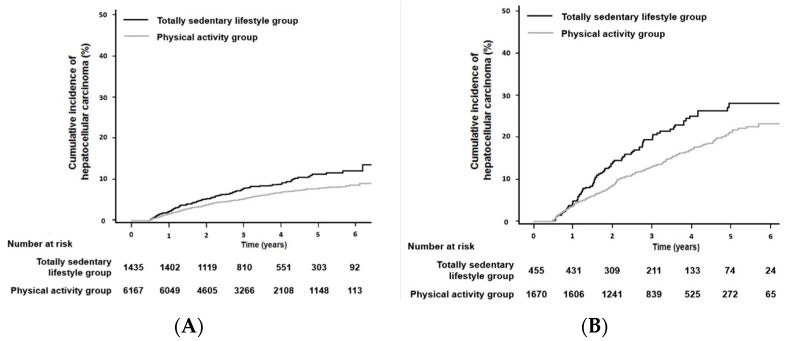
Kaplan–Meier estimate curve of HCC development in patients with chronic hepatitis B who were treated with entecavir or tenofovir. Kaplan–Meier estimate curve of HCC development for patients without liver cirrhosis (**A**) or patients with liver cirrhosis (**B**), according to physical activity (all, *p* < 0.001 by log-rank test).

**Figure 3:**
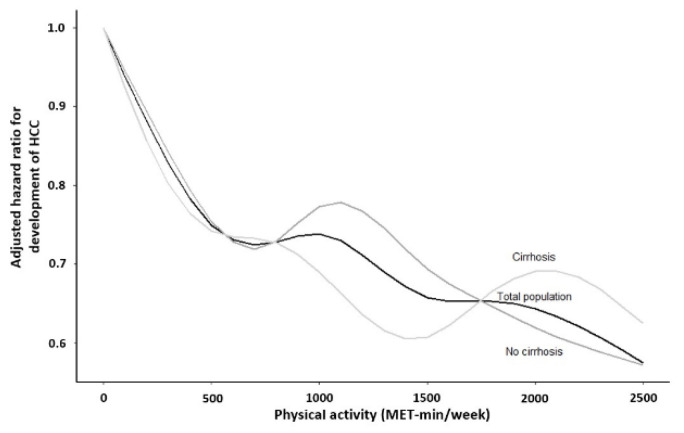
Nonlinear relationship between physical activity and the risk of HCC development according to the presence of liver cirrhosis.

In patients without cirrhosis or with cirrhosis, the results suggested that patients with a physical activity level of 1000–1500 MET-min/week were at a significantly lowest risk of HCC development compared to those with a totally sedentary lifestyle (aHR 0.66; 95% CI, 0.47–0.92; *p* = 0.02 and aHR 0.61; 95% CI, 0.40–0.93; *p* = 0.02, respectively; Table 2). The cumulative incidence of HCC development in patients with a totally sedentary lifestyle was significantly higher than in those engaging in physical activity, both in patients without and with cirrhosis (median value of 468 MET-min/week, IQR 174–834 MET-min/week; *p* < 0.001 by log-rank test in patients without cirrhosis; Figure 2A and median value of 435 MET-min/week, IQR 140–801 MET-min/week; *p* = 0.002 by log-rank test in patients with cirrhosis; Figure 2B, respectively). There was a nonlinear relationship between physical activity and the risk of HCC development according to the presence of liver cirrhosis (Figure 3). In patients with cirrhosis, the lowest risk of HCC development (a nadir) was found in patients with a physical activity level of 1000–1500 MET-min/week, compared to those with other amounts of physical activities.

### 3.3. Association of Physical Activity with HCC Development According to Subgroups of Age, Sex, Diabetes, and BMI at Baseline

To find out which patient groups can have a strong risk reduction of HCC development by physical activity, the association between amounts of physical activity and HCC risk was analyzed in various subgroups (Table 3).

When patients were divided into groups aged <60 years and ≥60 years, younger patients (<60 years) with physical activity levels of 500–1000 and 1000–1500 MET-min/week had a significantly lower risk of HCC development compared to those who had a totally sedentary lifestyle (aHR 0.75; 95% CI, 0.60–0.94; *p* = 0.01 and aHR 0.65; 95% CI, 0.48–0.88; *p* = 0.005, respectively). Older patients (≥60 years) with a physical activity level of 500–1000 MET-min/week had a significantly lower risk of HCC development compared to those who had a totally sedentary lifestyle (aHR 0.68; 95% CI, 0.47–0.99; *p* = 0.04).

In subgroup analyses according to sex, male patients with any amount of physical activity had a significantly lower risk of HCC development compared to those who had a totally sedentary lifestyle. Among various amounts of physical activity, male patients with a physical activity level of 1000–1500 MET-min/week had a significantly lower risk of HCC compared to those who had a totally sedentary lifestyle (aHR 0.63; 95% CI, 0.46–0.85; *p* = 0.003).

The results suggest that patients without diabetes who took part in any amount of physical activity had a significantly lower risk of HCC development compared to those who had a totally sedentary lifestyle. The findings suggest that among various amounts of physical activity, the patients without diabetes who had a physical activity level of 1000–1500 MET-min/week had a significantly lower risk of HCC compared to those who had a totally sedentary lifestyle (aHR 0.65; 95% CI, 0.50–0.86; *p* = 0.002). However, in patients with diabetes mellitus, there was no significant difference of HCC risk between patients with physical activity and those without it.

In subgroup analyses according to BMI (high BMI means BMI ≥ 25 kg/m^2^, low BMI means BMI < 25 kg/m^2^), the results suggested that patients with a high BMI and a physical activity level of 500–1000 and 1000–1500 MET-min/week had a significantly a lower risk of HCC compared to those who had a high BMI and a totally sedentary lifestyle (aHR 0.66; 95% CI, 0.48–0.90; *p* = 0.009 and aHR 0.64; 95% CI, 0.42–0.97; *p* = 0.03, respectively). Findings suggest that patients with a low BMI and a physical activity level of 1000–1500 MET-min/week had a significantly lower risk of HCC compared with those who had a low BMI and a totally sedentary lifestyle (aHR 0.66; 95% CI, 0.47–0.92; *p* = 0.01).

## 4. Discussion

In this study, the results suggest that physical activity of more than 500 MET-min/week was significantly associated with a lower risk of HCC development. Approximately half of the study population did not reach the recommended level of leisure-time physical activity (500 MET-min/week) [35] and particularly, 19.4% of total patients had a totally sedentary lifestyle. CHB patients with cirrhosis had patterns of physical activity similar to those of patients without cirrhosis. In particular, physical activity had more benefits to lower the risk of HCC development in younger, noncirrhotic, nondiabetic, overweight, and male patients.

Although the results suggest that HCC risk was the highest in those with a totally sedentary lifestyle and lowest in those with a physical activity level of 1000–1500 MET-min/week (a nadir) regardless of cirrhosis, the degree of relative risk reduction for HCC development was the highest, 18%, for those who had between 0 and 500 MET-min/week. This tended to be weakened above 500 MET-min/week: further risk reduction was 8% between patients with <500 MET-min/week and those with 500–1000 MET-min/week, and 10% between those with 500–1000 MET-min/week and those with 1000–1500 MET-min/week. This means that starting light intensity of leisure-time physical activity, even amounts of <500 MET-min/week, in patients who had a totally sedentary lifestyle, can be clinically important to prevent HCC development.

Contrary to our prediction, the findings in this study suggest that the risk of HCC was rather increased in patients with cirrhosis who had ≥1500 MET-min/week as compared with those who had 1000–1500 MET-min/week: the dose-response relationship extended up to 1500 MET-min/week. This suggests that cirrhotic patients who maintained vigorous physical activity did not benefit from exercise to prevent HCC as much as would be expected. One possible reason for this may be that a high level of physical activity can induce increased levels of serum testosterone [36], which can play a role in increasing the risk of HCC in patients with cirrhosis [37]. Androgens exert a stimulatory effect on HCC development [38], and it has been reported that HCC development can be attenuated after knocking down the androgen receptor expression in a mouse model [39].

In subgroup analyses of this study, there was a tendency that physical activity was more beneficial to lower the risk of HCC development in younger patients who did not have cirrhosis or diabetic mellitus. This reflects that early intervention of increasing physical activity in CHB patients treated with entecavir or tenofovir can be clinically important to prevent HCC development. In addition, this may mean that it might be important to start physical activity in the early phase of hepatic fibrosis in patients without cirrhosis, before the development of advanced fibrosis or cirrhosis. After cirrhosis development, preventive outcomes of physical activity on HCC development were decreased in patients with cirrhosis. Given that sarcopenia is independently associated with hepatic fibrosis in previous studies [40,41,42], physical activity can attenuate progression of sarcopenia [43,44] and thereby, it might show preventive outcomes of HCC development by suppression of hepatic fibrosis [45].

The median duration of treatment with entecavir or tenofovir from the start of treatment was 37.8 months (IQR, 23.7–41.9 months) in the no-HCC group and 19.8 months (IQR, 9.6–30.1 months) in the HCC group (*p* < 0.001). Because we enrolled patients who showed good compliance of >80% of MPRs of entecavir or tenofovir, treatment duration of entecavir or tenofovir was very close to the follow-up duration in each group: a mean difference of 1.3 months between duration of antiviral treatment and total follow-up duration. Although there was a significant difference of treatment duration between the no-HCC group and the HCC group, ratios of antiviral treatment duration and total follow-up duration were not significantly different between the no-HCC group (ratio = 0.97) and the HCC group (ratio = 0.98) (*p* = 0.70). A follow-up period in the HCC group was shorter than that in the no-HCC group because patients who developed HCC during the follow-up periods were censored in this study. Thus, treatment duration in the no-HCC group was shorter than that in the HCC group due to difference of follow-up duration between the two groups.

There were several limitations in this study. Recall bias is one of the major potential limitations. Information about physical activity depended on self-reported questionnaires administered during the individuals’ follow-up examinations. These questionnaires investigated lifestyle patterns during the previous week. Second, various types of physical activity occur throughout a day for diverse purposes [46]. Although only leisure-time physical activity was analyzed in this study, occupation, transportation, and household physical activities also contribute to total daily physical activity. Third, we cannot exclude the presence of unadjusted confounding factors because of the retrospective design of this study. For example, participants with a higher physical activity level may have paid more attention to disease prevention and a healthy diet, which may affect a better prognosis. Fourth, several assumptions were made while calculating the amount of physical activity. As the questionnaires focused primarily on aerobic physical activity, information on muscle-strengthening and bone-strengthening exercises was limited. Lastly, we did not suggest any experimental results explaining the mechanism of how physical activity can directly lower HCC risk in in vivo and in vitro studies. Caution is needed when interpreting that there may be a causal relationship between physical activity and HCC development in CHB patients of this study. Further studies focused on cellular signaling and animal studies should be performed to suggest a mechanism how physical activity can prevent HCC carcinogenesis.

In conclusion, this population-based study suggests that increasing physical activity has beneficial outcomes on HCC development in CHB patients treated with potent antiviral therapy such as entecavir or tenofovir.

## Figures and Tables

**Table 1:** Baseline characteristics of the total study population.

Variables	Total (*n* = 9727)	No HCC (*n* = 8918)	HCC (*n* = 809)	*p*-Value ^a^	No Cirrhosis (*n* = 7602)	Cirrhosis (*n* = 2125)	*p*-Value ^a^
Age, years	49 ± 10	49 ± 10	53 ± 10	<0.001	48 ± 10	54 ± 9	<0.001
Male, *n* (%)	6008 (61.8)	5438 (61.0)	570 (70.5)	<0.001	4665 (61.4)	1343 (63.2)	0.13
Cirrhosis, *n* (%)	2125 (21.8)	1788 (20.0)	337 (41.7)	<0.001	–	–	–
Body mass index, kg/m^2^	23.8 (21.7, 25.9)	23.7 (21.7, 25.9)	24.2 (21.9, 26.4)	0.002	23.9 (21.6, 25.9)	24.1 (22.0, 26.3)	<0.001
Diabetes mellitus, *n* (%)	404 (4.2)	321 (3.6)	83 (10.3)	<0.001	14 (0.2)	390 (18.4)	<0.001
Hypertension, *n* (%)	581 (6.0)	466 (5.2)	115 (14.2)	<0.001	13 (0.2)	568 (26.7)	<0.001
Significant alcohol consumption, *n* (%)	318 (3.3)	255 (2.9)	63 (7.8)	<0.001	239 (3.1)	79 (3.7)	0.21
Smoking				<0.001			0.31
Never, *n* (%)	5462 (56.2)	5077 (56.9)	385 (47.6)		4295 (56.5)	1167 (54.9)	
Previous, *n* (%)	1988 (20.4)	1819 (20.4)	169 (20.9)		1526 (20.1)	462 (21.7)	
Current, *n* (%)	2275 (23.4)	2020 (22.7)	255 (31.5)		1779 (23.4)	496 (23.3)	
Unknown, *n* (%)	2 (0.0)	2 (0.0)	0 (0.0)		2 (0.0)	0 (0.0)	
Laboratory findings							
AST, IU/L	51 (35, 83)	50 (34, 84)	54 (39, 76)	0.04	53 (36, 89)	45 (33, 64)	<0.001
ALT, IU/L	61 (37, 110)	62 (37, 113)	53 (35, 83)	<0.001	69 (40, 125)	44 (30, 67)	<0.001
GGT, IU/L	42 (25, 79)	40 (24, 76)	62 (35, 123)	<0.001	40 (24, 76)	48 (28, 91)	<0.001
Serum creatinine, mg/dL	0.9 (0.7, 1.0)	0.9 (0.7, 1.0)	0.9 (0.7, 1.0)	0.17	0.9 (0.7, 1.0)	0.9 (0.7, 1.0)	0.013
GFR, ml/min/1.73 m^2^	89 (78, 103)	89 (78, 103)	89 (78, 104)	0.91	89 (78, 103)	89 (77, 104)	0.35
Fasting blood glucose, mg/dL	93 (86, 102)	93 (85, 102)	95 (87, 106)	<0.001	92 (85, 101)	95 (87, 105)	<0.001
Total cholesterol, mg/dL	185 (163, 209)	186 (164, 209)	179 (157, 200)	<0.001	187 (165, 210)	179 (157, 201)	<0.001
HDL cholesterol, mg/dL	56 (47, 67)	56 (47, 67)	56 (47, 67)	0.67	56 (47, 67)	56 (46, 67)	0.06
LDL cholesterol, mg/dL	108 (88, 128)	108 (89, 129)	101 (83, 121)	<0.001	109 (89, 130)	103 (84, 122)	<0.001
Medication use							
Aspirin, *n* (%)	355 (3.6)	334 (3.7)	21 (2.6)	0.12	271 (3.6)	84 (4.0)	0.44
Statin, *n* (%)	962 (9.9)	908 (10.2)	54 (6.7)	0.002	795 (10.5)	167 (7.9)	<0.001
Leisure-time physical activity, MET-min/week	454 (174, 828)	480 (174, 834)	360 (0, 729)	<0.001	468 (174, 834)	435 (140, 801)	0.07
Totally sedentary, *n* (%)	1890 (19.4)	1676 (18.8)	214 (26.5)	<0.001	1435 (18.9)	455 (21.4)	0.10
Duration of antiviral treatment, months	37.7 (22.1, 53.4)	37.8 (23.7, 41.9)	19.8 (9.6, 30.1)	<0.001	38.4 (23.9, 52.8)	34.4 (20.2, 48.6)	<0.001

Values are expressed as the mean with standard deviation or median with IQR for continuous variables and frequency with proportion for categorical variables. Significant alcohol consumption means ≥210 g per week in men and ≥140 g per week in women. ^a^ *p* value estimated by χ^2^-test with Yates’ correction for categorical variables, and *t*-test or Wilcoxon rank-sum test for continuous variables. Abbreviations: ALT, alanine aminotransferase; AST, aspartate aminotransferase; GGT, gamma-glutamyl transferase; HCC, hepatocellular carcinoma; HDL, high-density lipoprotein; IQR, interquartile range; LDL, low-density lipoprotein; MET, metabolic equivalent of task.

**Table 2:** Leisure-time physical activity and the risk of HCC development stratified by the presence of liver cirrhosis.

Amount of Leisure-Time Physical Activity	HCC per 100 Person-Years	Multivariable-Adjusted ^a^	
		HR (95% CI)	*p*-Value
Total population			
Totally sedentary	3.35	Reference	
<500 MET-min/week	2.46	0.82 (0.68–0.99)	0.04
500–1000 MET-min/week	2.29	0.74 (0.61–0.89)	0.002
1000–1500 MET-min/week	1.96	0.64 (0.50–0.84)	0.001
≥1500 MET-min/week	2.40	0.70 (0.51–0.97)	0.03
No liver cirrhosis			
Totally sedentary	2.43	Reference	
<500 MET-min/week	1.84	0.83 (0.65–1.06)	0.13
500–1000 MET-min/week	1.67	0.72 (0.56–0.92)	0.009
1000–1500 MET-min/week	1.56	0.66 (0.47–0.92)	0.02
≥1500 MET-min/week	1.61	0.68 (0.44–1.06)	0.09
Liver cirrhosis			
Totally sedentary	6.61	Reference	
<500 MET-min/week	4.84	0.81 (0.60–1.08)	0.81
500–1000 MET-min/week	4.65	0.77 (0.57–1.03)	0.77
1000–1500 MET-min/week	3.45	0.61 (0.40–0.93)	0.02
≥1500 MET-min/week	5.16	0.76 (0.47–1.23)	0.76

Abbreviations: CI, confidence interval; HCC, hepatocellular carcinoma; HR, hazard ratio; MET, metabolic equivalent of task. ^a^ The multivariable-adjusted model was adjusted for significant variables in univariable analyses, including age, sex, cirrhosis (for total population), diabetes mellitus, hypertension, body mass index, smoking, significant alcohol drinking, AST, ALT, GGT, HDL-cholesterol, LDL-cholesterol, GFR, creatinine, fasting blood glucose, entecavir or tenofovir, aspirin (time-dependent variable), and statin (time-dependent variable). A competing risk analysis was also conducted: competing risk of liver transplant or death for HCC development.

**Table 3:** Leisure-time physical activity and the risk of HCC development stratified by specific subgroups.

Amount of Leisure-Time Physical Activity	HCC per 100 Person-Years	Multivariable-Adjusted ^a^		HCC per 100 Person-Years	Multivariable-Adjusted ^a^	
		HR (95% CI)	*p*-Value		HR (95% CI)	*p*-Value
Age	<60 years old			≥60 years old		
Totally sedentary	2.68	Reference		6.45	Reference	
<500 MET-min/week	2.10	0.81 (0.65–1.01)	0.06	4.99	0.81 (0.57–1.17)	0.26
500–1000 MET-min/week	2.00	0.75 (0.60–0.94)	0.01	4.14	0.68 (0.47–0.99)	0.04
1000–1500 MET-min/week	1.68	0.65 (0.48–0.88)	0.005	3.92	0.64 (0.39–1.05)	0.08
≥1500 MET-min/week	2.00	0.71 (0.47–1.06)	0.09	4.22	0.64 (0.36–1.13)	0.13
Sex	Male			Female		
Totally sedentary	4.21	Reference		2.33	Reference	
<500 MET-min/week	2.72	0.74 (0.59–0.93)	0.01	2.04	1.01 (0.73–1.39)	0.96
500–1000 MET-min/week	2.61	0.70 (0.56–0.88)	0.002	1.73	0.83 (0.59–1.18)	0.30
1000–1500 MET-min/week	2.24	0.63 (0.46–0.85)	0.003	1.41	0.70 (0.42–1.15)	0.16
≥ 1500 MET-min/week	2.74	0.64 (0.44–0.93)	0.02	1.69	0.84 (0.43–1.62)	0.60
Diabetes mellitus	No diabetes			Diabetes		
Totally sedentary	3.15	Reference		7.46	Reference	
<500 MET-min/week	2.34	0.81 (0.67–0.99)	0.03	5.99	0.89 (0.48–1.63)	0.70
500–1000 MET-min/week	2.07	0.70 (0.57–0.85)	0.001	8.38	1.21 (0.69–2.13)	0.51
1000–1500 MET-min/week	1.86	0.65 (0.50–0.86)	0.002	4.07	0.59 (0.25–1.37)	0.22
≥1500 MET-min/week	2.17	0.70 (0.50–0.98)	0.04	7.38	1.01 (0.42–2.40)	0.98
Body mass index (BMI)	BMI ≥ 25 kg/m^2^			BMI < 25 kg/m^2^		
Totally sedentary	4.02	Reference		3.00	Reference	
<500 MET-min/week	2.89	0.78 (0.58–1.05)	0.10	2.24	0.84 (0.66–1.07)	0.16
500–1000 MET-min/week	2.45	0.66 (0.48–0.90)	0.009	2.20	0.78 (0.61–1.00)	0.049
1000–1500 MET-min/week	2.42	0.64 (0.42–0.97)	0.03	1.72	0.66 (0.47–0.92)	0.01
≥1500 MET-min/week	2.47	0.64 (0.38–1.06)	0.08	2.36	0.72 (0.47–1.10)	0.13

Abbreviations: BMI, body mass index; CI, confidence interval; HCC, hepatocellular carcinoma; HR, hazard ratio; MET, metabolic equivalent of task.^a^ The multivariable-adjusted model was adjusted for significant variables in univariable analyses, including age, sex, cirrhosis, diabetes mellitus, hypertension, body mass index, smoking, significant alcohol drinking, AST, ALT, GGT, HDL-cholesterol, LDL-cholesterol, GFR, creatinine, fasting blood glucose, entecavir or tenofovir, aspirin (time-dependent variable), and statin (time-dependent variable). A competing risk analysis was also conducted: competing risk of liver transplant or death for HCC development.

## Data Availability

Data used in this study are not available due to the NHIS policy for data.

## Supplementary Materials

The following are available online at https://www.mdpi.com/article/10.3390/cancers13143424/s1, Figure S1: Physical activity of patients with and without cirrhosis. Figure S2: Comparison of physical activity between male and female. Table S1: Definition of diagnosis. Table S2: Multivariable analysis for HCC development in total study population.

## Abbreviations

ALT, alanine aminotransferase; AST, aspartate aminotransferase; BMI, body mass index; CHB, chronic hepatitis B; CI, confidence interval; GGT, gamma-glutamyl transferase; HBV, hepatitis B virus; HCC, hepatocellular carcinoma; HR, hazard ratio; ICD, International Classification of Diseases; NHIS, National Health Insurance Service.
