# Supplementary material for: Association of Physical Activity with the Risk of Hepatocellular Carcinoma in Patients with Chronic Hepatitis B
Source: Cancers (Basel). 2021 Jul 8;13(14):3424. doi: 10.3390/cancers13143424 (PMC8306325; doi:10.3390/cancers13143424)

Supplementary Materials

Association of physical activity with the risk of hepatocellular carcinoma in patients with chronic hepatitis B

Supplementary Table S1. Definition of diagnosis

Supplementary Table S2. Multivariable analysis for HCC development in total study population

Supplementary Fig. S1. Physical activity of patients with and without cirrhosis

Supplementary Fig. S2. Comparison of physical activity between male and female

Supplementary Table S1. Definition of diagnosis

Variable	Definition
**Exclusion criteria**	
HCV coinfection	ICD-10 code: B18.2
HIV coinfection	ICD-10 codes: B20-B24
Cancer	ICD-10 codes: C00-C97
**Covariables**	
Cirrhosis	ICD-10 code: K7290, K7291, K74, K740, K7400, K7401, K7402, K7409
Diabetes mellitus	ICD-10 codes: E100, E1000, E1001, E1002, E1003, E1008, E101, E1010, E1011, E1012, E1018, E102, E1020, E1021, E1022, E1028, E103, E1031, E1032, E1033, E1034, E1038, E104, E1040, E1041, E1042, E1048, E105, E1050, E1051, E1058, E106, E1060, E1061, E1062, E1063, E1064, E1068, E107, E1070, E1071, E1072, E1078, E108, E109, E11, E110, E1100, E1101, E1102, E1103, E1108, E111, E1110, E1111, E1112, E1118, E112, E1120, E1121, E1122, E1128, E113, E1131, E1132, E1133, E1134, E1138, E114, E1140, E1141, E1142, E1148, E115, E1150, E1151, E1158, E116, E1160, E1161, E1162, E1163, E1164, E1168, E117, E1170, E1171, E1172, E1178, E118, E119, E14, E140, E1400, E1401, E1402, E1403, E1408, E141, E1410, E1411, E1412, E1418, E142, E1420, E1421, E1422, E1428, E143, E1431, E1432, E1433, E1434, E1438, E144, E1440, E1441, E1442, E1448, E145, E1450, E1451, E1458, E146, E1460, E1461, E1462, E1463, E1464, E1468, E147, E1470, E1471, E1472, E1478, E148, E149.
Hypertension	ICD-10 codes: I10, I101, I109, I11, I110, I119, I12, I120, I129, I13, I130, I131, I132, I139, I15, I150, I151, I152, I1520, I1521, I1522, I1528, I158, I1580, I1588, I159
**Outcomes**	
HCC	ICD-10 code C22 with the special cancer claim code V193

Supplementary Table S2. Multivariable analysis for HCC development in total study population (n=9,727)

	HR	95% CI	*P* value
Physical activity			
Totally sedentary	Reference		
< 500 MET-min/week	0.82	0.68–0.99	0.039
500–1,000 MET-min/week	0.74	0.61–0.89	0.002
1,000–1,500 MET-min/week	0.64	0.50–0.84	0.001
≥ 1,500 MET-min/week	0.70	0.51–0.97	0.032
Antiviral therapy			
Entecavir	Reference		
Tenofovir	0.89	0.77–1.02	0.101
Age	1.04	1.03–1.05	<0.001
Sex (female vs male)	0.67	0.55–0.82	<0.001
Diabetes mellitus	1.36	1.03–1.80	0.032
Hypertension	1.39	1.09–1.77	0.009
Body mass index	1.03	1.01–1.06	0.004
Smoking			
Never	Reference		
Previous	1.01	0.82–1.25	0.904
Current	1.32	1.09–1.62	0.006
Significant alcohol consumption	1.33	0.99–1.79	0.057

AST	1.000	0.998–1.001	0.779
ALT	0.998	0.996–0.999	0.005
GGT	1.002	1.002–1.003	<0.001
Fasting blood glucose	1.000	0.998–1.003	0.687
LDL cholesterol	0.995	0.993–0.997	<0.001

Abbreviations: ALT, Alanine aminotransferase; AST, aspartate aminotransferase; CI, confidence interval; GGT, gamma-glutamyl transferase; HCC, hepatocellular carcinoma; HR, hazard ratio; LDL, low-density lipoprotein; MET, metabolic equivalent of task. .

Supplementary Fig. S1. The proportions of physical activity classified into 5 categories stratified by the presence of cirrhosis. There was no significant difference of the proportions of physical activity between the two groups (*P*=0.10)

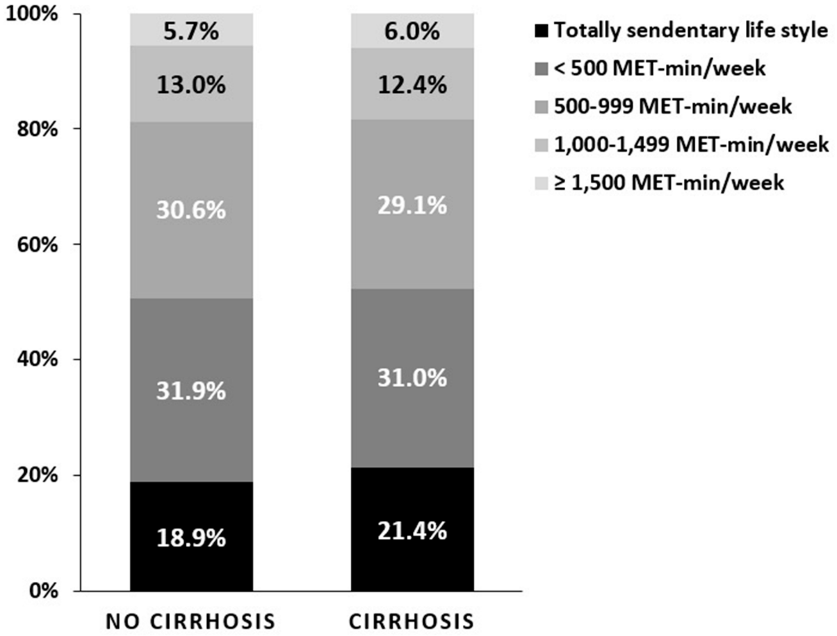

Supplementary Fig. S2. The proportions of physical activity classified into 5 categories stratified by sex. There was significant difference of the proportions of physical activity between the two groups (*P*<0.001): male patients had more physical activity than female patients.

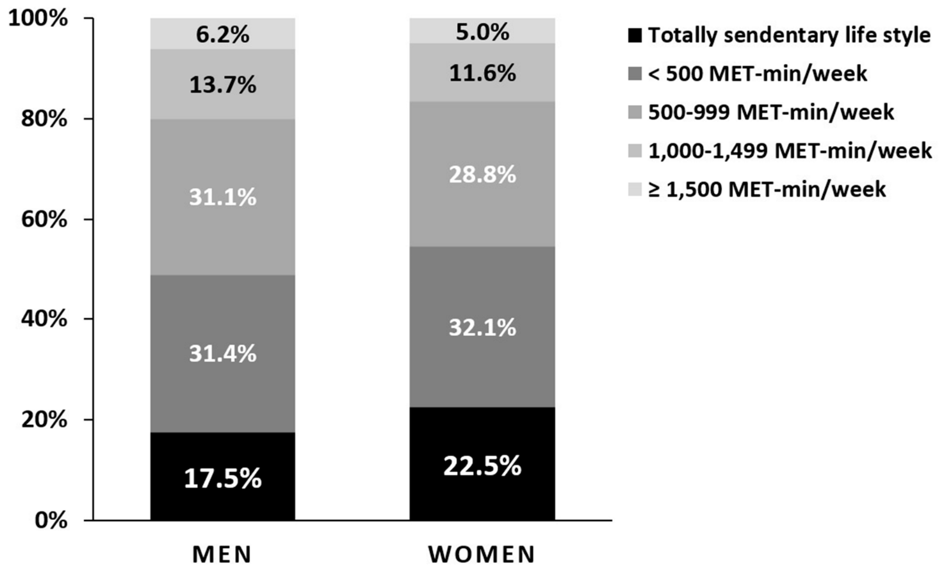

Supplement: Supplementary file 1 [file cancers-13-03424-s001.zip › cancers-1251193-supplementary.pdf]
